# Inhibitors of the Cellular Trafficking of Ricin

**DOI:** 10.3390/toxins4010015

**Published:** 2012-01-06

**Authors:** Julien Barbier, Céline Bouclier, Ludger Johannes, Daniel Gillet

**Affiliations:** 1 Laboratory of Molecular Toxinology and Biotechnology (LTMB), Molecular engineering of proteins (SIMOPRO), Life Sciences Division (DSV), Institute of Biology and Technology Saclay (iBiTec-S), French Alternative Energies and Atomic Energy Commission (CEA), F-91191 Gif sur Yvette, France; Email: julien.barbier@cea.fr (J.B.); celine.bouclier@gmail.com (C.B.); 2 Institut Curie/CNRS UMR144, Centre de Recherche, Traffic, Signaling, and Delivery Laboratory, 26 rue d’Ulm, F-75248 Paris Cedex 05, France; Email: ludger.johannes@curie.fr

**Keywords:** ricin, chemical genetics, retrograde transport, small-molecule inhibitor

## Abstract

Throughout the last decade, efforts to identify and develop effective inhibitors of the ricin toxin have focused on targeting its *N*-glycosidase activity. Alternatively, molecules disrupting intracellular trafficking have been shown to block ricin toxicity. Several research teams have recently developed high-throughput phenotypic screens for small molecules acting on the intracellular targets required for entry of ricin into cells. These screens have identified inhibitory compounds that can protect cells, and sometimes even animals against ricin. We review these newly discovered cellular inhibitors of ricin intoxication, discuss the advantages and drawbacks of chemical-genetics approaches, and address the issues to be resolved so that the therapeutic development of these small-molecule compounds can progress.

## 1. Why Target Cellular Components? A Chemical Genetics Approach

Ricin toxin is a lectin produced in the seed of the plant *Ricinus communis*. This plant is used for ornamentation, but also has a high industrial value for the oil contained in the seed. The oil enters in the composition of brake fluids, soaps, shampoos, cosmetics and paints. However, the hydrophilic toxin remains in the crushed seeds from which it can be easily purified. Its lethal dose is a few µg per kg when injected in mice and between 1 and 20 mg if ingested in humans. Public authorities consider ricin as a potential bio-crime and bio-terrorist weapon for which there is absolutely no specific antidote. In addition, ricin has been used for the design of immunotoxins against tumor cells, although non-specific toxicity prevented those to reach approval for cancer therapy. To counter the threat displayed by the plant toxin ricin, several therapeutic approaches have been developed. One approach is the use of vaccination to elicit the production of neutralizing antibodies that inactivate ricin *in vivo* [1,2]. However, it is unrealistic to perform a mass vaccination program to protect whole populations against ricin. Another approach is to design new antitoxins. However, although neutralizing antibodies are effective in animal models [3,4], they are of limited use in clinical practice due to their cost and limited therapeutic window. So far, the catalytic activity of the enzymatic moiety of ricin (the A chain, termed RTA) has been the target of choice for inhibition by small-molecule compounds such as transition-state analogues [5] based on pterin and purine scaffolds [6,7,8], or nucleic acid ligands [9,10,11]. Such enzymatic inhibitors have been identified by virtual screens or by *in vitro* selection [8,11,12,13]. Although active in enzymatic tests, they usually fail to protect cells or animals against a ricin challenge. There is only one published report of an anti-ricin A-chain RNA aptamer (31RA) that protects cells against ricin exposure [10].

Alternatively, few molecules altering intracellular trafficking have been shown to block ricin toxicity. However, the dramatic effect of these compounds on the integrity of the Golgi apparatus does not allow their development for therapy. Screening for small-molecule inhibitors of cellular targets is a complementary means of identifying bioactive compounds against ricin. This approach is often termed chemical genetics, and focuses on the identification of new pharmacological targets and chemical scaffolds that show the desired activity on cells. RNAi-based screening, another possible strategy to identify cell proteins involved in ricin toxicity, will not be discussed here. Cell-based assays do not exclusively aim to identify enzymatic inhibitors. Other targetable pathways, which are investigated, include: binding to cell-surface receptors, internalization, intracellular trafficking, dissociation of the catalytic RTA from the receptor-binding B chain (termed RTB), and retro-translocation of RTA across the ER membrane to the cytosol. Another advantage of cell-based assays is the ability to monitor the toxicity and cell permeability of inhibitors in the same system used for the screening process.

**Figure 1:**
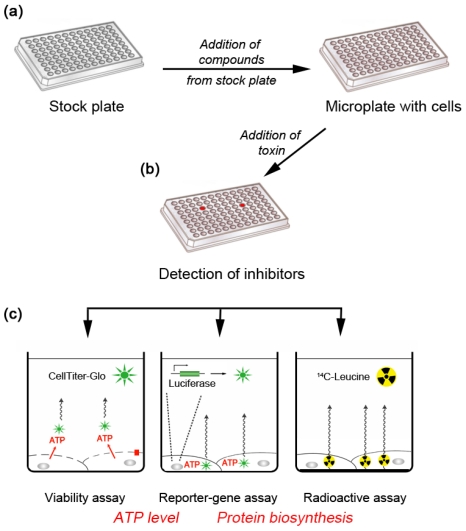
High-throughput cell-based assays. These phenotypic assays measure the effects of small-molecule compounds on cellular cytotoxicity induced by ricin. (**a**) Chemical compounds are distributed into microplates for screening. A small volume of each compound is then added to a well of a microplate seeded with cells. (**b**) After addition of toxin and incubation, one of several possible methods is used to assess the effects of each compound on the toxin-mediated inhibition of protein biosynthesis. (**c**) The CellTiter-Glo luminescent cell-viability assay quantifies ATP, which signals the presence of metabolically active cells. Luminescent signal is generated by the luciferase (green star) reaction after cell lysis and is proportional to the amount of ATP present [14]. In luciferasereporter-gene assays, a modified luciferase is constitutively transcribed, and enzyme activity is used as a measure of ongoing protein biosynthesis. Inhibition of protein biosynthesis thus leads to diminish luciferase translation, and as existing luciferase protein is degraded there is a proportional decrease in light output [21]. In the third method of assessment, the inhibitory effect of ricin on protein biosynthesis in intact cells is measured through the incorporation of radioactive amino acids into neosynthesized polypeptides. Intact cells are able to concentrate the amino acid that strikes the incorporated scintillant molecules present in the bottom of each well (bold line) in the microplate [15].

Cell-based high-throughput screening (HTS) studies have been used by research teams to identify inhibitors that can protect cells against toxins such as ricin and Shiga toxin [14,15,16]. Ricin and the bacterial Shiga toxin share several characteristics. They have one moiety (the B chain or B-subunit) that binds to their respective cellular receptors (glycoproteins and glycolipids for ricin; the glycosphingolipid Gb3 for Shiga toxins), while another moiety (the A chain or A-subunit) enters the cytosol and inactivates protein synthesis. Both toxins are transported in a retrograde manner from the plasma membrane to the endoplasmic reticulum (ER) [17], before translocation to the cytosol where they enzymatically inactivate the 28S RNA of the 60S ribosomal subunit (reviewed in [17,18,19,20]. It is therefore likely that inhibitors acting on the intracellular routing of Shiga toxins will also interrupt the trafficking of ricin. This review on ricin will thus also discuss compounds mentioned in Section 2 that have been described as Shiga-toxin inhibitors. Phenotypic screening approaches based on inhibition of protein biosynthesis in mammalian cells have provided a robust platform for analyzing libraries in chemical-genetic studies, and have been used to identify ricin inhibitors (Figure 1). In an initial study by Saenz *et al*., a luciferase-based assay was used to screen 14,400 molecules and identified two compounds that protected monkey Vero cells from the cytotoxic effects of Shiga toxin and ricin [16]. The methodology involved transfection of cells with cDNA encoding a destabilized luciferase with a short half-life. Thus, cells treated with toxin showed a rapid decrease in luciferase activity. The two compounds identified showed a marked potency and selectivity against intracellular toxin transport.

Finally, Stechmann *et al*. have used a protein biosynthesis assay to screen the effects of 16,480 molecules from a commercial library of drug-like compounds on A549 human epithelial pulmonary cells [15]. Several ricin inhibitors were identified, and two of these were studied in further detail. Both inhibitors selectively block retrograde toxin trafficking at the early endosome/*trans*-Golgi network (TGN) interface. One compound protected mice exposed to a lethal dose of ricin administered by nasal instillation. 

## 2. Inhibitors of Retrograde Transport Toxins that Act Inside Cells

### 2.1. Compounds that Target an Identified Host Target

Characterization of molecules that protect cells against ricin by blocking its intracellular trafficking has revealed compounds that often alter the morphology of the Golgi apparatus. This limits their therapeutic interest due to potentially strong side effects.

**Figure 2:**
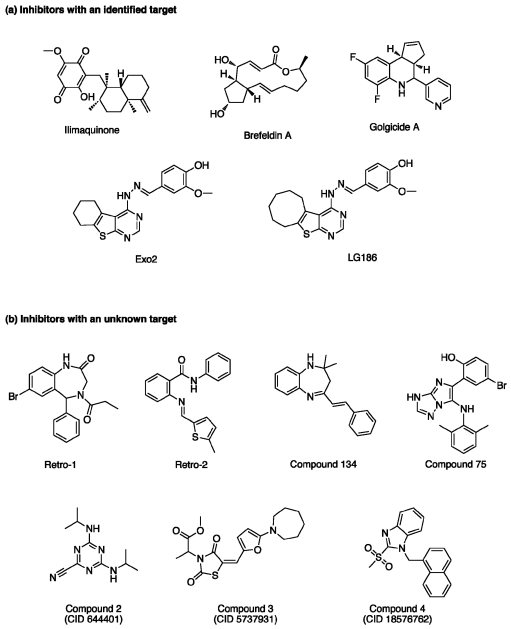
Chemical structures of the known cellular inhibitors of ricin and Shiga toxins. (**a**) Structures of inhibitors with a known molecular target; (**b**) Structure of inhibitors identified by cellular high-throughput screening whose cellular targets are currently unidentified. The CID number is the *Chemical IDentifier* for molecular structures in PubChem. References for the molecules are given in the text.

**Ilimaquinone** (IQ, MW: 358.5, Figure 2a) is a sesquiterpenoid quinone metabolite isolated from marine sponges. IQ inhibits the cytotoxicity of ricin on Vero cells in a dose-dependent manner [22]. This inhibition is reversed when IQ is removed from the culture medium. IQ causes breakdown of the Golgi apparatus and its fragmentation into small vesicular structures [23,24]. This is likely because IQ interacts with enzymes of the activated methyl cycle such as S-adenosylmethionine synthetase, S-adenosylhomocysteinase, and methyl transferases [25]. Although some authors have suggested that IQ has an effect on small GTPases, no direct evidence for this property has been reported.

**Figure 3:**
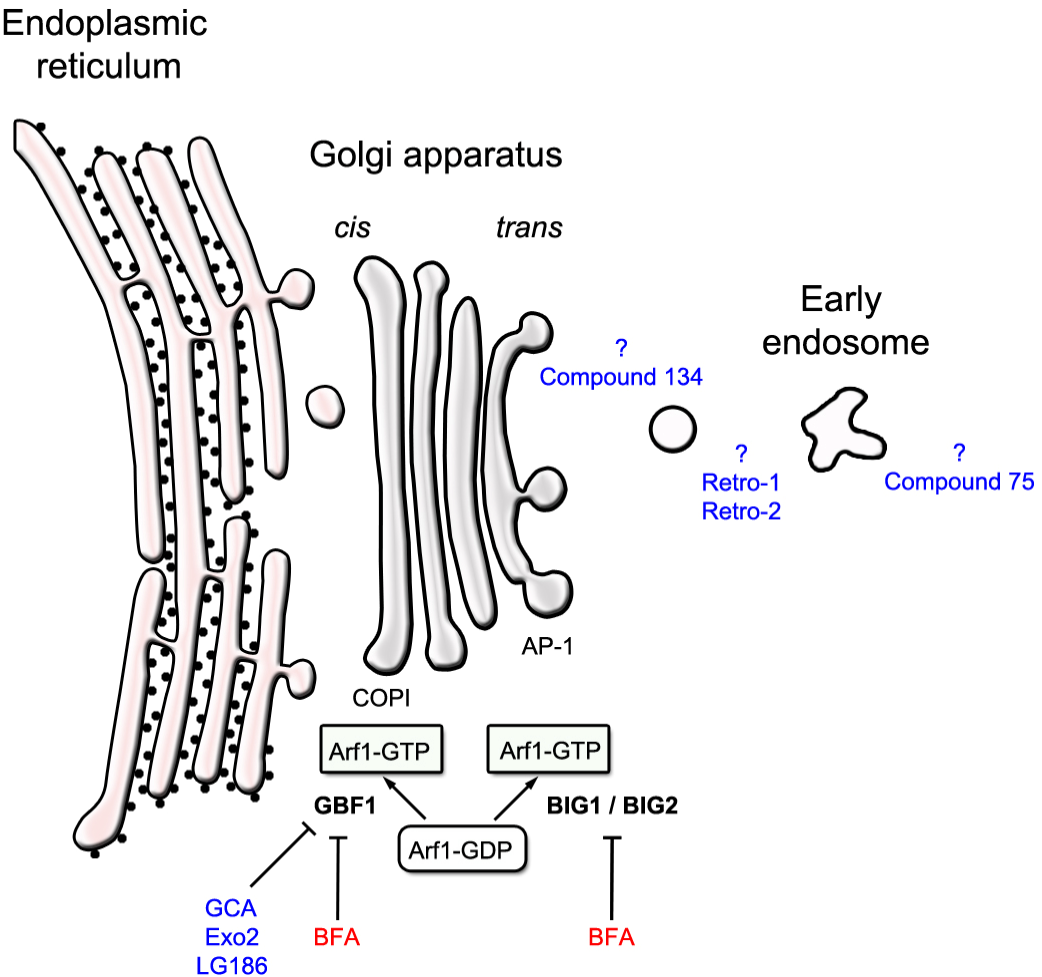
Cellular targets of some inhibitors of ricin and/or Shiga toxins.

**Brefeldin A** (BFA, MW: 280.4, Figure 2a) is an isoprenoid fungal metabolite that inhibits ricin toxicity *in vitro* and protects cells from the cytotoxic effects of ricin and Shiga toxin [26,27,28]. BFA disrupts the structure and function of the Golgi apparatus, and strongly impairs intracellular protein transport and secretion [29]. Although BFA protects a number of cell lines against ricin, some cell lines such as the MDCK and PtK2 kidney epithelial cell lines, are sensitized to ricin [30]. These differential effects of BFA are probably due to variations in the structural organization of the Golgi apparatus among the different cell lines. BFA inhibits the activation and function of the ADP-ribosylation factor (Arf) family by inhibiting specific guanine nucleotide exchange factors (GEFs) [31]. GEFs regulate Arf GTPase by accelerating the nucleotide exchange from its inactive GDP-bound form to its active GTP-bound form, which can interact with effectors [32,33]. Golgi-localized Arf1 is present in eukaryotic cells and regulates anterograde and retrograde traffic [34,35]. Arf1 recruits the coatomer complex at the *cis*-Golgi network to assemble COPI-coated vesicles, whereas clathrin adaptor proteins are recruited at the *trans*-Golgi network and on endosomes. The *cis*-Golgi localized GEF, Golgi BFA resistance factor 1 (GBF1), and the *trans*-Golgi localized GEFs, Brefeldin A-inhibited GEP 1 (BIG1) and Brefeldin A-inhibited GEP 2 (BIG2) which share a Sec7 domain required for guanine nucleotide exchange, are sensitive to BFA (Figure 3). Activation of Arf1 by GBF1 mediates COPI coat recruitment and enables transport of vesicles between the ER and the Golgi apparatus [36]. Activation of BIG1 and BIG2 leads to Arf1-recruitment of clathrin adaptors, such as AP-1, AP-3, and AP-4, which mediate transport between the Golgi and endosomal compartments [37]. More precisely, the BFA targets the complex between Arf-GDP and the catalytic domain (the sec7 domain) of an ArfGEF at the beginning of the exchange reaction. BFA binds to the interface between Arf-GDP and ArfGEF and blocks the complex in an abortive conformation that cannot proceed to GDP/GTP exchange [32]. BFA is by far one of the best-characterized GEF inhibitors; this characterization has taken approximately 40 years since its discovery as a natural product. Understanding the mechanism of action of BFA has led to the general concept of interfacial inhibition. This refers to inhibitors that trap macromolecules in transition states with their partners near interacting interfaces in dead-end complexes which are unable to complete their biological role. Furthermore, the resistance to BFA in some cell lines, such as canine MDCK, has been related to a single amino-acid substitution (M832L) within the Sec7 domain of GBF1, which modifies its interaction with BFA [32,38,39]. Nevertheless, the high toxicity of BFA precludes its use as a pharmaceutical treatment for ricin poisoning.

The compound **Golgicide A** (GCA, MW: 284.3) was identified by Saenz *et al*. in a HTS study of small molecules that inhibited the effects of bacterial toxins on host cells [39]. When used at a concentration of 10 µM, GCA strongly protects Vero cells from Shiga toxin. GCA is a potent and reversible inhibitor of the *cis*-Golgi ArfGEF, GBF1, but has no effect on BIG1 and BIG2 (*cf*. Figure 3). GCA also causes complete dispersal of the *medial*-Golgi marker, giantin and the *cis*-Golgi marker, GM130. As observed with BFA, mutagenesis and molecular-modeling studies show that GCA binds at the interface of Arf1 and GBF1 Sec7 domain [39]. Inhibition of GBF1 results in the dissociation of the COPI vesicle coat from the Golgi membranes and subsequent disassembly of the Golgi and *trans*-Golgi networks.

**Exo2** (MW: 354.4) was first identified in chemical genetics studies of inhibitors of the secretory pathway [40,41]. This compound rapidly blocks anterograde traffic from the ER to the Golgi apparatus, and selectively disrupts the Golgi apparatus. Exo2 was then used as a pharmaceutical tool to study the function of the Golgi apparatus in retrograde toxin trafficking [42,43]. These studies showed that Exo 2 has no effect on trafficking of cholera toxin from the cell surface to the ER [42], but significantly inhibits the delivery of Shiga-toxin to the ER [43]. Similar to BFA, Exo2 disrupts the morphology of the TGN [43]. However, in contrast with BFA, Exo2 does not induce tubulation and fusion of the TGN and endosomal membranes [43]. Furthermore, treatment with Exo2 dissociates COPI, but leaves AP-1 intact. This may be because Exo2 selectively inhibits GBF1 as a primary target among the ArfGEFs (*cf*. Figure 3). However, even if Exo2 inhibits Arf-GEF function, the other phenotypic changes induced by Exo2 may be unrelated to its effects on GBF1 [44].

The Exo2 derivative, **LG186** (MW: 382.5), was synthesized to enhance selectivity for GBF1 [44]. Compared with Exo2, LG186 has a cyclo-octenyl ring instead of a cyclohexenyl ring within a tricyclic structure, but is otherwise identical (*cf*. Figure 2). Although BFA, GCA, and Exo2 do not perturb the Golgi complex in MDCK cells, LG186 removes COPI from vesicle membranes and induces a collapse of the Golgi complex [44]. Molecular-modeling studies suggest that the cyclo-octenyl ring of LG186 compensates for the loss of interaction of the M832L residue of GBF1 with GCA, BFA, and Exo2 in MDCK cells. However, LG186 appears to have a residual inhibition of other ArfGEFs. Other derivatives of Exo2 have also been designed to conserve the protective effects of Exo2 against Shiga toxin and to decrease its cytotoxicity [45,46]. Exo2 and its derivatives have therefore provided more selective tools than BFA to decipher membrane trafficking in mammalian cells.

### 2.2. Compounds with Unknown Molecular Targets

Two compounds, named **75** (MW: 398.3) and **134** (MW: 276.4) which protect Vero cells from the cytotoxic effects of ricin and Shiga toxin, have been identified in a cellular HTS study by Saenz *et al*. [16] (*cf*. Figure 2b). The protective effects of these compounds results from their disruption of intracellular transport at distinct steps along the retrograde toxin-trafficking pathway. The maximal protective effects against cytotoxicity are observed at a concentration of 50 µM for compound 75, and 100 µM for compound 134. Compound 75 protects Vero cells against Shiga toxin, ricin, as well as diphtheria toxin (DT), whereas compound 134 is inactive against DT. DT is released into the cytosol from endosomes after a translocation step at low pH. The lack of protection against DT by compound 134 thus suggests that this inhibitor acts only after the early endosome stage. In contrast, compound 75 is likely to act during the very early events of endocytosis. Fluorescence microscopy analysis revealed the presence of STxB in the early endosomes of cells treated with compound 75 [16]. In contrast, STxB reaches the perinuclear recycling endosomes of cells treated with compound 134. Neither compound affects the binding or endocytosis of STxB, nor do they inhibit anterograde transport between ER-to-Golgi. However, in contrast with compound 134, compound 75 partially blocks secretion from the TGN. Both compounds induce morphological changes to the Golgi apparatus, and their effects are reversible. Sulfation assays (for further details, see [47]) demonstrated that the trafficking of toxins to the TGN is reduced by 53% with compound 75, and by 28% with compound 134. Compound 75 thus appears to inhibit transport at an early stage of endocytosis, whereas compound 134 appears to inhibit transport at a post-recycling endosome stage (*cf*. Figure 3). However, the targets of these two compounds are yet to be identified. It is nevertheless unlikely that either compound can be developed for therapeutic use due to their effects on the integrity of the Golgi apparatus, which is an essential compartment for cellular homeostasis.

Wahome *et al*. [14] have identified four small-molecule inhibitors with significant dose-dependent anti-ricin activity and EC_50_ values ranging from 23 to 62 µM. These small-molecule inhibitors also have a low to moderate cytotoxicity. One compound appears to be an enzymatic inhibitor of RTA. The three others, named **compound 2** (CID 644401; MW: 220.3), **compound 3** (CID 57337931; MW: 378.4), and **compound 4** (CID 18576762; MW: 336.4) (*cf*. Figure 2b), most likely interfere indirectly with ricin cytotoxicity by acting on cellular processes rather than on the toxin itself. However, no in-depth characterization of these compounds has been performed, and putative targets are yet to be identified.

Stechmann and co-workers [15] have recently identified two compounds, **Retro-1** (MW: 373.2) and **Retro-2** (MW: 320.1) (*cf*. Figure 2b), which protect human cells against ricin and Shiga toxins. Morphological and quantitative biochemical trafficking assays show that Retro-1 and Retro-2 inhibit retrograde toxin transport between early endosomes and the TGN/Golgi complex (*cf*. Figure 3). Using a STxB variant, developed as a biochemical tool [47], cells treated with Retro-1 or Retro-2 showed an 80% reduction of retrograde transport to the Golgi complex. Electron microscopy and confocal microscopy experiments demonstrated that STxB accumulates in the early endosomes of cells treated with Retro-1 or Retro-2. However, Retro-1 and Retro-2 are highly selective compared with other inhibitors, and thus lack toxicity. Neither compound has any effect on the TGN46 endogenous retrograde cargo proteins or on the cation-independent mannose 6-phosphate receptor (CI-MPR). Furthermore, the structural and functional integrity of all organelles tested, such as the Golgi complex, the early and late endosomes, and the ER, is preserved in cells exposed to Retro-1 and Retro-2. Other intracellular trafficking events that intersect with the early endosomes/TGN interface, such as endocytosis, endocytic recycling, and the biosynthetic/secretory pathways, are not affected by treatment with these compounds. Retro-1 and Retro-2 do not show any additive or synergistic effect, and it is therefore likely that they share a common target that is yet to be identified. Retrograde transport at the early endosomes/TGN interface is regulated by a large number of trafficking factors [17]. The location of 26 of these factors was studied in cells treated with Retro-1 and Retro-2. Only syntaxin-5 was relocalized within minutes of compound treatment, suggesting that this SNARE protein is a direct or indirect target of Retro-1 and Retro-2. Furthermore, Retro-2 completely protects mice exposed to a lethal ricin challenge, and is therefore a promising compound for further development as a drug acting on retrograde toxins. As Retro-2 has to be given prior to ricin exposure, the drug delivery pathways and pharmaceutical formulations need to be improved to maximize the benefits of this inhibitor. Nevertheless, the retrograde transport route is a potential therapeutic target in the development of broad-spectrum antibiotic compounds against pathogens or pathogenic products that use this pathway to gain entry into cells.

## 3. Identifying Intracellular Protein-Binding Targets of Ricin Inhibitors

The potential of phenotypic screens for the discovery of useful compounds against ricin toxicity is promising. However, this method requires the development of a structure-based rational drug-design to identify molecular targets. Identifying intracellular protein-binding targets of small-molecules is an integral part of chemical genetics. Identification of these targets during the early stages of drug discovery can help to address issues such as selectivity, toxicity, and off-target pharmacology. Indeed, hits can target any protein, which makes this identification process difficult and probably the most rate-limiting step in the development of useful compounds. The possibility of success in target identification greatly increases with binding affinity. However, much of the difficulty arises from weakly binding interactions between compounds identified in HTS and their cellular targets. Although nanomolar or lower binding constants make target identification feasible, in most cases inhibitors from screens have micro-molar activities [14,15,16]. Another difficulty in understanding of the mode of action of inhibitors is related to the use of cellular phenotypes as readouts, which often involve intricate biological processes and interactions between various pathways. This level of complexity can present major challenges when characterizing the targets and mode of action of inhibitors.

The classical approach to identify targets of inhibitors is based on affinity chromatography. This technique requires immobilization of the inhibitor (bait compound) to a solid support such as magnetic beads or beads for affinity chromatography, which can be achieved through a linker arm. Another possibility is introducing a reactive group (e.g., a photo-affinity label) that can be activated to crosslink with target proteins. Bait compounds can also have a detection tag such as a fluorescent moiety, radioactive tracer (e.g., ^14^C, or ^3^H), or biotin for non-covalent immobilization on streptavidin-coated beads. For target identification, it is essential to achieve these chemical modifications without interfering with the activity of the bioactive molecules. Analyzing cellular protection with libraries of compounds targeted against ricin is a key step to study structure-activity relationships. Rapid development of these hits into more potent molecules without the use of fastidious chemistry is a major technical hurdle. Furthermore, affinity chromatography will often reveal non-obvious interacting partners. Once an inhibitor has been immobilized, cell lysates or protein extracts are then passed through a column of inhibitor-decorated beads. Proteins that bind very tightly are retained, while non-binding proteins are washed away. Bound proteins can be eluted, separated by SDS-PAGE, and identified by mass spectrometry. As mentioned above, the main difficulty of this process is the inability to isolate proteins with a low binding affinity, because high-stringency washes are required to minimize contamination with non-specific proteins. This problem can be circumvented by using a covalent cross-linking between the inhibitor and the target protein. Another key determinant of the success or failure of affinity chromatography is the abundance of the target protein(s). It is far more difficult for a low-abundance protein to be detected above background level, unless the identification scheme includes an enrichment step that enhances sensitivity in the MS read-out. To achieve protein identification, it is important to work with the same cell line as that used for HTS. We have observed that some ricin inhibitors identified by HTS have varying degrees of potency in their protection of different cell lines. This might reflect variations in the expression levels of target-proteins among these different cell lines. Many variations of affinity-based target identification strategies exist. These include, stable isotope labeling with amino acids in cell culture (SILAC), [48]) including genetic approaches by phage display, three-hybrid screens [49,50], and drug affinity responsive target stability (DARTS) [51]. New ways of creating high-affinity probes from weak inhibitors have also been reported, and these are based on linking two low-affinity compounds onto one scaffold to exploit the effect of avidity [52,53].

## 4. Conclusions

The studies that we have reviewed demonstrate the use of cell-based HTS to identify new chemical entities that protect cells and mice against ricin. This approach was derived from the observation that molecules disrupting the Golgi apparatus, although improper for therapeutic use, protected cells against intoxication by ricin and other toxins. The most promising leads target the retrograde pathway. Simple and robust tests have been used to screen large libraries; over 100,000 small-compounds have been tested so far, resulting in the discovery of less than 10 chemical entities with robust activity against the cytotoxic effects of ricin. Although the size of the compound libraries and the number of high-throughput screens targeting retrograde toxins may continue to increase, the difficulty is to select the most promising candidates for further exploration. HTS is a very costly process (estimated $0.1 to $10 per compound), and the coverage of relevant chemical space needs to be improved by selecting new chemical libraries for screens. Besides libraries of bioactive compounds (drugs), most chemical libraries used for cell-based HTS come from two different sources: natural resources and synthetic chemical libraries. The former has the advantage of a higher structural diversity, whereas the latter corresponds to “drug-like” compounds in accordance with the rules of Lipinski [54]. Although these rules are useful to assess the risk profile of a drug candidate that is entering development, they do not necessarily define the properties of a good lead. Thus, the “quality” of libraries needs to be improved, and other filters may be used to select collections for future screens. Furthermore, a virtual screening approach could be of interest if the identified compounds described in this review interact with new cellular targets. This may save resources and reduce the experimental work involved by suggesting limited sets of molecules to be tested.
